# Effectiveness and safety of prolotherapy injections for management of lower limb tendinopathy and fasciopathy: a systematic review

**DOI:** 10.1186/s13047-015-0114-5

**Published:** 2015-10-20

**Authors:** Lane M. Sanderson, Alan Bryant

**Affiliations:** School of Surgery, University of Western Australia, Perth, Australia; M422 UWA Podiatric Medicine, 35 Stirling Highway, Crawley, WA 6009 Australia

## Abstract

**Introduction:**

The aim of this review was to identify and evaluate existing research to determine the clinical effectiveness and safety of prolotherapy injections for treatment of lower limb tendinopathy and fasciopathy.

**Review:**

Nine databases were searched (Medline, Science Direct, AMED, Australian Medical Index, APAIS-Health, ATSIhealth, EMBASE, Web of Science, OneSearch) without language, publication or data restrictions for all relevant articles between January 1960 and September 2014. All prospective randomised and non-randomised trials, cohort studies, case-series, cross-sectional studies and controlled trials assessing the effectiveness of one or more prolotherapy injections for tendinopathy or fasciopathy at or below the superior aspect of the tibia/fibula were included. Methodological quality of studies was determined using a modified evaluation tool developed by the Cochrane Musculoskeletal Injuries Group. Data analysis was carried out to determine the mean change of outcome measure scores from baseline to final follow-up for trials with no comparative group, and for randomised controlled trials, standardised mean differences between intervention groups were calculated. Pooled SMD data were calculated where possible to determine the statistical heterogeneity and overall effect for short-, intermediate- and long-term data. Adverse events were also reported.

Two hundred and three studies were identified, eight of which met the inclusion criteria. These were then grouped according to tendinopathy or fasciopathy being treated with prolotherapy injections: Achilles tendinopathy, plantar fasciopathy and Osgood-Schlatter disease. The methodological quality of the eight included studies was generally poor, particularly in regards to allocation concealment, intention to treat analysis and blinding procedures. Results of the analysis provide limited support for the hypothesis that prolotherapy is effective in both reducing pain and improving function for lower limb tendinopathy and fasciopathy, with no study reporting a mean negative or non-significant outcome following prolotherapy injection. The analysis also suggests prolotherapy injections provide equal or superior short-, intermediate- and long-term results to alternative treatment modalities, including eccentric loading exercises forAchilles tendinopathy, platelet-rich plasma for plantar fasciopathy and usual care or lignocaine injections for Osgood-Schlatter disease. No adverse events following prolotherapy injections were reported in any study in this review.

**Conclusions:**

The conclusions of this review were derived from the best available scientific evidence. It is intended that the results of this study will assist clinical decision-making by practitioners. The results of this review found limited evidence that prolotherapy injections are a safe and effective treatment for Achilles tendinopathy, plantar fasciopathy and Osgood-Schlatter disease, however more robust research using large, methodologically-sound randomised controlled trials is required to substantiate these findings.

**Electronic supplementary material:**

The online version of this article (doi:10.1186/s13047-015-0114-5) contains supplementary material, which is available to authorized users.

## Introduction

Lower-limb pain has been identified as a risk factor for locomotor disability, impaired balance, increased risk of falling and reduced health-related quality of life [1]. Causes of lower limb pain are varied, including but not limited to, osteoarthritis, previous trauma, inflammatory and overuse injuries [1, 2]. Current non-surgical treatments for lower-limb musculoskeletal pain include activity modification, physical therapy, therapeutic ultrasound, extracorporeal shock wave therapy, dry needling, strapping, foot orthoses, mobilisations, corticosteroid injection, platelet-rich plasma injection and prolotherapy injection [1, 2].

Prolotherapy, as defined by Webster’s *Third New International Dictionary*, is “the rehabilitation of an incompetent structure, such as a ligament or tendon, by the induced proliferation of new cells” [3]. It is an increasingly popular regenerative injection technique for treatment of a wide range of musculoskeletal pathologies [3]. Most prolotherapy research focuses on its use in sprained and degenerated ligaments, however it is also reported to be an effective treatment for concomitant pain and joint dysfunctions caused by laxity of damaged periarticular dense connective tissues, and can be applied to other damaged dense connective tissues structures such as tendons and entheses [4–12]. The most commonly used prolotherapy substance reported in current literature is dextrose, a simple aldostic monosaccharide synonymous with glucose that is readily available in Australia and the United Kingdom in various concentrations [12].

The mechanism of action, safety and efficacy of dextrose prolotherapy is well represented in current literature. It is hypothesised that injection of dextrose causes local cell necrosis as a direct result of osmotic shock [5]. It is hypothesised that this method of intentional small scale “therapeutic trauma” at the injection site initiates the body’s wound healing cascade of inflammation, granulation tissue formation, and matrix formation and remodelling [11–13]. As stresses are placed on the new tissue, the collagen fibres align in the direction of the stress [11–13]. The effect of dextrose on the biomechanical properties of human tendons and the histological analysis are as yet unknown [5]. According to deChelli et al. [14], Rabago et al. [15] and Rabago et al. [16], the use of prolotherapy injections has been predominantly guided by anecdotal clinical success. Randomised controlled trials have demonstrated Level I - III evidence (as per the National Health and Medical Research Council designations of levels of evidence [17]) for injection of 10 to 25 % dextrose in areas of damaged ligament, tendon, and cartilage in adults to manage finger osteoarthritis, knee osteoarthritis, lateral epicondylosis, sacroiliac joint pain [18–21]. Several case-series reports of participants with anterior cruciate ligament laxity, coccygodynia, abdominal tendinosis and chronic back, hip adductor, ankle, foot and first metatarsophalangeal pain have also reported in favour of prolotherapy, however the poor methodological quality of these studies significantly reduces the weight of these findings [22–29].

Prolotherapy has been reported with increasing frequency in recent literature for treatment of chronic, painful lower-limb tendon and fascia overuse conditions [30–37]. These were formerly associated with the suffix ‘–itis’ but this has changed to ‘-osis’ or ‘-opathy’ to reflect existing underlying pathology [2]. Most prominently in the lower-limb, these include Achilles tendinopathy and plantar fasciopathy [30–36]. Recent evidence also suggests that prolotherapy injections may be an effective treatment for painful traction apophysitis pathologies through treatment of associated tendinopathy; such as patellar tendinopathy associated with Osgood-Schlatter disease [37]. In this situation, it is speculated that prolotherapy may reverse the neovascularisation accompanying tendinopathy and fasciopathy and stimulate the release of multiple growth factors that aid in tissue repair [37].

In examining the safety of prolotherapy, Dorman conducted a retrospective analysis of 494,845 participants who all underwent prolotherapy injections for various pathologies [38]. Dorman reported that very few adverse effects were found following prolotherapy injections and of these, the majority of serious complications were overwhelmingly in areas of the body outside the lower limb [38]. These included 29 reports of pneumothoraces, 24 reports of allergic reactions (none classified as serious) and 12 other reports of hospitalisation [38]. It should be noted that significant recall bias was present in Dorman’s study as the practitioners surveyed were reporting on complications in their patients entirely from memory [38]. Many of the procedures in Dorman’s study also used injections of overt sclerosants no longer used (such as zinc sulphate, which reportedly resulted in the death of a patient who received a lower-back injection in 1959), rather than the simple local anaesthetics and dextrose that are most commonly used today [38, 39].

Prolotherapy has been suggested to be a safe and effective treatment for a range of axial and upper limb pathologies. The proposed benefits of prolotherapy injections in musculoskeletal pathologies are based on the premise that clinical improvement is derived from inducing inflammation to stimulate the body’s wound healing cascade, enabling the repair of damaged tissue and new collagen formation, along with a reduction in associated neovascularity. The aim of this study was to review the clinical effectiveness and safety of prolotherapy injections for treatment of lower limb tendinopathy and fasciopathy.

## Review

### Methods

The question asked in this systematic review is placed under the category of ‘interventions’— as described by the National Health and Medical Research Council (NHMRC) handbook on conducting systematic reviews [40]. In preparing this review, the NHMRC criteria for reviewing ‘interventions’ was used as a guide.

### Selection of studies for inclusion

The types of studies considered for this review included both randomised and non-randomised trials, cohort studies, case-series, cross-sectional studies and controlled trials. Exclusion criteria included retrospective studies without a comparative control group, case reports, systematic reviews, review articles and general articles. While the authors would have preferred to include only randomised or quasi-randomised controlled trials in this review this would have significantly reduced the number of studies included in the analysis. All participants presenting for treatment of tendon or fascia pathology at or below the superior margin of tibia/fibula were included. Studies involving non-human participants (i.e. rats) were excluded. All age groups and both sexes were included.

For the purpose of this review, all treatments that were described as ‘prolotherapy’ were considered. Trials undertaken where no specific treatment was reported were excluded. The concentrations of dextrose and additional local anaesthetic solutions and concentrations varied between studies, and are described in detail in the ‘Description of studies’ section. Outcomes sought included both objective and subjective outcomes.

Objective data included in the analyses were:

(i) Sonographic measurements (tendon thickness, size of hypoechoic region and intratendinous tear size).
(ii) Post-intervention adverse events (total number of complications from each intervention was analysed).
(iii) Post-intervention return to activity (number of participants able to return to a particular activity).

Subjective outcome measures included in the analyses were:

(i) Post-intervention pain levels (the number of participants remaining in pain, or the pain level measured on a 0–100 mm visual analogue scale was used for the purposes of this review).
(ii) Post-intervention satisfaction levels (the number of participants dissatisfied or the level of dissatisfaction on a 0–100 mm visual analogue scale at the end of the trial was used).
(iii) Pathology or location-specific outcome measures (this includes the Victorian Institute of Sport Assessment-Achilles (VISA-A), Foot Function Index (FFI), stiffness and limited range of motion).

### Search methods for identification of studies

This systematic review was reported in accordance with Cochrane Collaboration and NHMRC guidelines [40, 41]. One reviewer (LS) conducted all screening of identified studies according to the pre-determined inclusion criteria. The Cochrane library was searched initially to ensure no systematic reviews had previously been conducted on this topic. Nine databases were systematically reviewed (Medline, Science Direct, AMED, Australian Medical Index, APAIS-Health, ATSIhealth, EMBASE, Web of Science, OneSearch) without language, publication or data restrictions for all relevant articles from January 1960 to September 2014, with the search keywords and synonyms of: Prolotherapy AND (tendonosis OR paratendonitis OR tendonitis OR tenosynovitis OR tendinopathy OR fasciitis OR fasciopathy OR foot OR ankle OR leg). Titles and abstracts were screened initially and those found to be irrelevant were excluded prior to screening full-text manuscripts.

Citations within obtained articles were hand-searched and scrutinised to identify additional studies. The relevant databases on the above list were searched for completed published, unpublished and ongoing unpublished studies. Conference proceedings were examined using the Index to Scientific and Technical Conference Proceedings [42]. Attempts to access the grey literature were made using the Health Management Information Consortium database, and System for Information on Grey Literature. The Australian New Zealand Clinical Trials Registry, National Institutes of Health Inventory of Clinical Trials and Studies, as well as the International Standard Randomised Controlled Trial Number database to ensure no other trials similarly assessing prolotherapy were commenced but never published. Attempts to access articles written in languages other than English were made using the Virtual Health Library and foreign language articles with abstracts were assessed. All relevant review articles and systematic reviews were also analysed to ensure no other relevant studies were missed.

### Quality assessment

An assessment of methodological quality was undertaken by one assessor (LS) using an evaluation tool developed by the Cochrane Musculoskeletal Injuries Group, which was modified for use in this systematic review. The following aspects of internal and external validity were considered:

A. *Was the assigned treatment adequately concealed prior to allocation?*
  2 = method did not allow disclosure of assignment
  1 = small but possible chance of disclosure of assignment or unclear
  0 = quasi-randomised or open-list/table
B. *Were the outcomes of participants who withdrew described and included in the analysis (intention to treat)?*
  2 = withdrawals well described and accounted for in analysis
  1 = withdrawals described and analysis not possible
  0 = no mention, inadequate, or obvious differences and no adjustments
C. *Were the outcome assessors blind to treatment status?*
  2 = effective action taken to blind assessors
  1 = small or moderate chance of unblinding assessors
  0 = not mentioned or not possible
D. *Were the participants blind to assignment status after allocation?*
  2 = effective action taken to blind participants
  1 = small or moderate chance of unblinding of participants
  0 = not possible, not mentioned (unless double-blind), or possible but not done
E. *Were important baseline characteristics reported and comparable?*
  The principal confounders were considered to be age and sex
  2 = good comparability of groups, or confounding adjusted for in analysis
  1 = confounding small, mentioned but not adjusted for
  0 = large potential for confounding, or not discussed
F. *Were care programs, other than the trial options, identical?*
  2 = care programs clearly identical
  1 = clear but trivial differences
  0 = not mentioned or clear and important differences in care programs
G. *Were the inclusion and exclusion criteria clearly defined?*
  2 = clearly defined
  1 = inadequately defined
  0 = not defined
H. *Were the interventions clearly defined?*
  2 = clearly defined interventions are applied with a standardised protocol
  1 = clearly defined interventions are applied but the application protocol is not standardised.
  0 = intervention and/or application protocol are poorly or not defined
I. *Were the outcome measures used clearly defined?*
  A general grading was given for all outcome measures used since the number and type varied between trials.
  2 = clearly defined
  1 = inadequately defined
  0 = not defined
  *Studies were also ranked by quality of allocation concealment (Cochrane score A, B, or C).*
  A = adequate
  B = unclear
  C = inadequate

### Data analysis

SPSS software version 17.0 (SPSS Inc, Chicago USA) was used to derive summary statistics. The standardised mean difference (SMD; difference in mean effects between groups divided by the pooled standard deviation) was calculated for studies with a control group or multiple interventions. If the difference in mean effects between groups was not available, the SMD was calculated from the post-intervention mean scores and corresponding standard deviation. The authors of one study, Yelland et al. [34], were directly contacted for visual analogue score (VAS) pain data which was collected but not included in their publication, as this was required to calculate the SMD of this study. Point estimates of effect were statistically significant when the 95 % confidence interval (CI) did not cross 0 for the SMD. Results favoured prolotherapy when the SMD was positive, and favoured the alternate intervention/control when the SMD was negative. SMD values of 0.0–0.5 were defined as a small effect, 0.5–0.8 a medium effect, and more than 0.8 a large effect. These were calculated for short-term (4 weeks, range 0–12), intermediate-term (26 weeks, 13–26) and long-term (52 weeks, ≥ 52) data. Pooled SMD data were also calculated where possible to determine the statistical heterogeneity and overall effect for short-, intermediate- and long-term data. Results were found to be statistically significant when *P* ≤ 0.05. Overall effects were determined using a random effects model if heterogeneity existed (*P* < 0.10), and a fixed effect model if the data were homogenous.

## Results

**Fig. 1:**
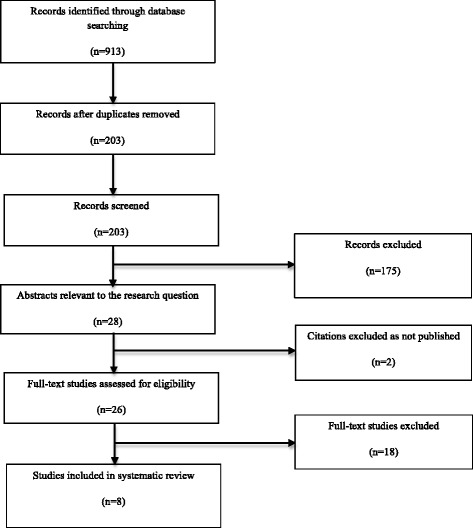
Quorum flowchart of the reviewing process

A total of 203 studies were identified through electronic search. Following the review of titles and abstracts, 26 studies were extracted for full review and finally, 8 studies were considered appropriate for inclusion (Additional file 1). The reasons for the exclusion of studies are available in Additional file 2. A flow diagram, as described by Moher et al. [43], is presented in Fig. 1 and highlights the study selection process. The included studies were assigned to one of three categories, based on the following comparisons: prolotherapy injection for Achilles tendinopathy, prolotherapy injection for plantar fasciopathy, and prolotherapy injection for Osgood-Schlatter Disease. Five studies were included in the Achilles tendinopathy section, two studies in the plantar fasciopathy section and one study in the Osgood-Schlatter Disease section of the review. All studies that met the inclusion criteria reported dextrose as the primary prolotherapy agent. See Table 1 for characteristics of included studies.

**Table 1:** Characteristics of included studies

Reference	Title	Objective	Study design	Population characteristics	Intervention/s	Outcome measures	Exclusion criteria
Prolotherapy injection for Achilles tendinopathy							
Lyftogt [30]	Prolotherapy and Achilles tendinopathy: A prospective pilot study of an old treatment.	To assess the clinical effectiveness of prolotherapy in the treatment of AT in a general medical/sports medicine setting with three-month follow-up; and a postulated positive relationship between initial VAS scores and number of treatments.	Prospective case series.	Location: New Zealand.	*Intervention:*	Pain results were monitored with individual prolotherapy recovergrams which were compiled in a study recovergram. A satisfaction survey was also performed.	Insertional AT.
				Sex: 4 female, 12 male.	1 mL subcutaneous prolotherapy, dextrose 20 %/lignocaine 0.1 %; at weekly intervals where possible.		
				Mean age: 48.0.			
Lyftogt [31]	Subcutaneous prolotherapy for Achilles tendinopathy: The best solution?	Reporting on treatment of chronic midportion AT with subcutaneous prolotherapy.	Prospective case series.	Location: New Zealand.	*Intervention*:	Follow up was conducted by an independent party with a standard questionnaire assessing VAS pain scores and overall satisfaction.	Not defined.
				Sex: 59 female, 85 male.	1 mL subcutaneous prolotherapy, three different dextrose/local anaesthetic regimens; at weekly intervals where possible.		
				Mean age: 48.0.			
Maxwell et al. [32]	Sonographically guided intratendinous injection of hyperosmolar dextrose to treat chronic tendinosis of the Achilles tendon: a pilot study.	Reporting on treatment of chronic AT with injections of hyperosmolar dextrose under sonographic guidance, to induce an inflammatory reaction and initiate a wound-healing cascade and subsequent collagen synthesis.	Prospective case series.	Location: Canada.	*Intervention*:	Improvement in VAS1, VAS2 and VAS3; satisfaction rating, as well as sonographic measurements of tendon thickness, size of hypoechoic region and intratendinous tear size.	Acute Achilles tendonitis or symptoms associated with acute trauma, surgery or interventional procedures in 3 months prior to trial.
				Sex: 11 female, 25 male.	≤2 mL prolotherapy, dextrose 25 %/lignocaine 1 %; every 6 weeks until either the symptoms resolved or no improvement.		
				Mean age: 54.0.			
Ryan et al. [33]	Favourable outcomes after sonographically guided intratendinous injection of hyperosmolar dextrose for chronic insertional and midportion Achilles tendinosis.	To report on changes in the short-term sonographic appearance at two year follow-up for pain outcomes in a large population with chronic AT who underwent sonographically guided injections of hyperosmolar dextrose.	Prospective Case series.	Location: Canada.	*Intervention*:	Improvement in VAS1, VAS2 and VAS3; as well as sonographic measurements of tendon thickness, size of hypoechoic region and intratendinous tear size.	Not defined.
				Sex: 41 female, 58 male.	≤2 mL prolotherapy, dextrose 25 %/lignocaine 1 %; every 6 weeks until either the symptoms resolved or no improvement.		
				Mean age: 54.0.			
Yelland et al. [34]	Prolotherapy injections and eccentric loading exercises for painful Achilles tendinosis: a randomised trial.	To compare the effectiveness of eccentric loading exercises (ELE); based on Alfredson protocol, with prolotherapy used alone and in combination for painful AT.	Single-blinded randomised controlled trial.	Location: Australia.	*Intervention*:	Improvement in VISA-A, pain stiffness and limitation of activity scores, satisfaction rating and treatment costs.	Previous steroid or prolotherapy or surgery to affected tendon, previous completion of >50 % of the Achilles ELE protocol, and allergies or medical conditions that may limit completion of treatments.
				Sex: not defined, 43 total participants.	≤5 mL dextrose 20 %/ lignocaine 0.1 %/ ropivacaine 0.1 % for 4–12 treatments (*n* = 15); vs *Comparator a)*: Combination of prolotherapy plus ELE (*n* = 14); vs *Comparator b)*: ELE (*n* = 14).		
				Mean age: 46.7.			
Prolotherapy injection for Plantar fasciopathy							
Kim et al. [35]	Autologous platelet-rich plasma versus dextrose prolotherapy for the treatment of chronic recalcitrant plantar fasciitis.	To determine the efficacy of autologous PRP compared with prolotherapy in participants with chronic recalcitrant plantar PF.	Single-blinded randomised controlled trial.	Location: Korea.	*Intervention*:	Pain, disability and activity limitation subscales, measured by means of the FFI.	Local steroid injections within 6 months or nonsteroidal anti-inflammatory drugs within 1 week prior to randomisation, active bilateral PF, or previous surgery for PF.
				Number: 10 female, 11 male.	2 mL dextrose 15 %/lignocaine 1.25 % (*n* = 11); vs *Comparator*: 2 mL of autologous PRP (*n* = 10). Two injections into the plantar fascia at interval of 2 weeks.		
				Mean age: 37.0.			
Ryan [36]	Sonographically guided intratendinous injections of hyperosmplar dextrose/lignocaine: a pilot study for the treatment of chronic plantar fasciitis.	To report on the effectiveness of sonographically guided injections of hyperosmolar dextrose at reducing the pain associated with chronic PF.	Prospective case series.	Location: Canada.	*Intervention*:	VAS1, VAS2 and VAS3, foot function index (FFI) were recorded at baseline and at final treatment consultation (post-test).	Acute plantar foot pain or symptoms associated with acute trauma. Surgery or interventional procedures within previous 6-months.
				Number: 17 female, 3 male.	≤2 mL dextrose 25 %/ lignocaine 1 %; injected sonographic guidance; 6-week intervals.		
				Mean age: 51.2.			
Prolotherapy injection for Osgood-Schlatter Disease							
Topol [37]	Hyperosmolar dextrose injection for recalcitrant Osgood-Schlatter disease.	To examine prolotheapy versus lignocaine injection versus supervised usual care to reduce sport alteration and sport-related symptoms in adolescent athletes with OSD.	Double-blinded randomised controlled trial.	Location: Argentina, USA.	*Intervention*:	The Nirschl pain phase scale (NPPS) was used to assess Unaltered sport (NPPS <4) and asymptomatic sport (NPPS = 0) were the threshold goals.	Not defined.
				Sex: 3 female, 51 male.	≥2 mL dextrose 12.5 %/lignocaine 1 % solution (*n* = 21); vs *Comparator a)*:≥2 mL lignocaine 1 % solution (*n* = 22); vs *Comparator b)*: Supervised standard therapy (*n* = 22).		
				Mean age: 13.3.			

*AT* Achilles tendinopathy, *VAS* visual analogue scale, *VAS1* pain during rest, *VAS2* pain during normal daily activity, *VAS3* pain during or after sporting activity, *ELE* eccentric loading exercises, *VISA*-*A* Victorian institute of sports assessment-Achilles, *PRP* platelet-rich plasma, *PF* plantar fasciopathy, *OSD* Osgood-Schlatter disease

### Quality assessment of included studies

The methodological quality scores for the eight included studies are shown in Table 2. The maximum possible score per study is 18 and each study was also graded for allocation concealment (A - concealed assignment, B - possible disclosure/unclear, C - disclosure likely).

**Table 2:** Quality of assessment for included trials

Study	Pathology	A	B	C	D	E	F	G	H	I	Total/18	Quality assessment	Allocation concealment
Kim et al. [35]	PF	1	1	0	0	2	2	2	2	2	12	Moderate	B
Lyftogt [30]	AT	0	0	0	0	0	0	1	2	2	5	Poor	C
Lyftogt [31]	AT	0	0	0	0	0	0	1	1	2	4	Poor	C
Maxwell et al. [32]	AT	0	1	0	0	0	2	2	2	2	9	Moderate	C
Ryan et al. [36]	PF	0	2	0	0	0	2	2	1	2	9	Moderate	C
Ryan et al. [33]	AT	0	1	0	0	0	2	1	1	2	7	Moderate	C
Topol et al. [37]	OSD	1	2	1	1	1	2	2	2	2	14	Good	B
Yelland et al. [34]	AT	2	2	2	0	0	2	2	2	2	14	Good	A

*PF* Plantar fasciopathy, *AT* Achilles tendinopathy, *OSD* Osgood-Schlatter disease, *A* concealed assignment, *B* withdrawals described, intention to treat analysis, *C* assessors blinded, *D* participant blinded, *E* groups comparable at entry, *F* identical care programs, *G* inclusions/exclusions defined, *H* interventions defined, *I* outcomes defined

The methodological quality of the studies in this review was generally poor. Allocation concealment (the procedure for protecting the randomisation process so that the treatment to be allocated is not known before the participants enter into the study) was poorly conducted in the majority of studies analysed in this review. The trials by Topol et al. [37] and Kim et al. [35] were two exceptions, however concealment was implied but not defined in both studies. All studies scored poorly for participant blinding, which is particularly difficult in injection intervention trials, with Topol et al. [37] the only study providing any reference of participant blinding. Only Yelland et al. [34] reported an adequate method of allocation concealment. In general, the selection of outcome measures was clinically appropriate and the duration of surveillance was adequate. The blinding of assessors was attempted in only two trials, although it is recognised that it may be difficult to blind an assessor when there may be evidence of the procedure being undertaken from post-injection signs. Many studies also failed to conduct, or provide data for, intention to treat analysis.

Yelland et al. [34] and Topol et al. [37] were the only studies with a ‘good’ overall quality assessment (see Table 2), with Yelland et al. [34] the only study in which adequate evidence of concealed allocation was provided, stating the randomisation schedule was generated and administered by telephone independently by the NHMRC Clinical Trials Centre in Sydney, Australia. Topol et al. [37] reported use of a random numbers table for assignment to each intervention group and stated that this was blinded to the participants, guardian and treating/evaluating physician; however, again, no specific mention was made of how this allocation was concealed. Kim et al. [35] used alternation of participants as a method of randomisation but did not define the method of concealment.

All other studies used a prospective single intervention case-series methodology, thus no randomisation or allocation concealment was carried out, with resulting ‘poor’ to ‘moderate’ quality assessment overall scores. Although all studies had reasonable scores regarding the definition and usefulness of outcome measurements (item I), they were generally very poor in quality regarding intention to treat analysis (item B) and blinding procedures (items C, D), although this may not have been possible in several of the studies.

### Study characteristics

There were three randomised controlled trials and five prospective case-series studies identified, the characteristics of which are presented in Table 1. Lyftogt [30] conducted a prospective case-series of 16 participants with non-insertional Achilles tendinopathy, to evaluate the clinical effectiveness of prolotherapy for relieving painful symptoms. Subcutaneous prolotherapy injections of 1 mL were performed weekly where possible with final follow-up at 3 months post-final treatment. Lyftogt [31] carried out a significantly larger study with similar methods 2 years later and used three different prolotherapy regimens: (i) dextrose 20 %/lignocaine 0.1 %, (ii) dextrose 30 %/lignocaine 0.1 %/ropivacaine 0.1 % and (iii) dextrose 40 %/ropivacaine 0.1 % for 87, 31 and 26 participants respectively. In this study, Lyftogt’s [31] final follow-up was undertaken independently after a mean period of 20 months. Both of Lyftogt’s studies involved mainly male participants and treated only midportion Achilles tendinopathy [30, 31].

Maxwell et al. [32] conducted a prospective case-series assessing pain, satisfaction and sonographic changes following sonographically-guided hyperosmolar dextrose injections in 36 participants (25 men and 11 women) with chronic Achilles tendinopathy. VAS pain during rest, VAS pain during normal daily activity and VAS pain during or after sporting activity scores and sonographic assessments were recorded 6 weeks after final treatment. In addition, a telephone interview with each study participant was performed a mean of 12 months (range 4.5–28 months) after the last treatment to assess the medium- to long-term efficacy of dextrose injection therapy. Ryan et al. [33] built on evidence presented in the study by Maxwell et al. [32] in a prospective case-series published 3 years later. Ninety-nine participants (58 men and 48 women) with painful midportion (86) and insertional (22) Achilles tendinopathy pain received the same prolotherapy regimen, outcome measures and treatment schedule as described by Maxwell et al. [32], however improvements were made on the length of follow-up with a mean final follow-up time of 28.6 months.

**Fig. 2:**
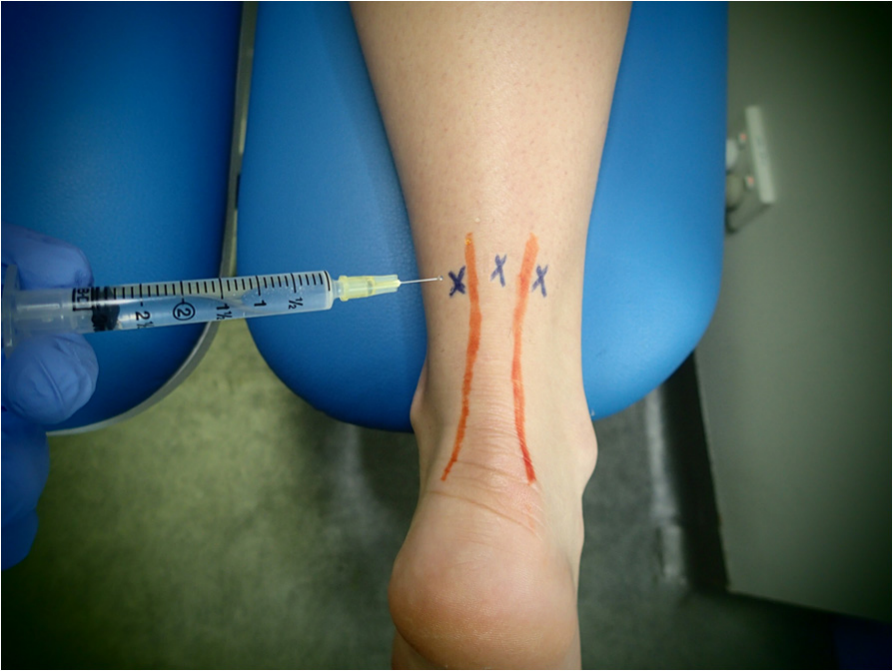
Posterior photograph of right lower leg showing injection points most commonly used by Yelland et al. [34] for management of Achilles tendinopathy. The ‘X’ markings represent the anteromedial, posterior midline and anterolateral margins of the tendon, with *orange lines* demarking the Achilles tendon

Yelland et al. [34] compared weekly prolotherapy injections with eccentric loading exercises and a combination of eccentric loading exercises plus prolotherapy injections in 43 participants with painful Achilles tendinopathy. The participants were randomly allocated using a computer-generated randomisation schedule to receive prolotherapy injections alone (15 participants), eccentric loading exercisesalone (14 participants) or a combination therapy of prolotherapy and eccentric loading exercises (14 participants). Between 0.5 and 1 mL of solution was injected at each tender point, most commonly the anterolateral and anteromedial margins of the tendon and on the most posterior aspect of the tendon 2–7 cm from the calcaneal attachment, as illustrated in Fig. 2. A maximum total of 5 mL of solution was used, with the number of treatments determined by the time it took to reach a pain-free activity or until the participant requested to cease treatment. Outcome assessments of VISA-A, VAS pain, stiffness and limitation of activity scores, satisfaction rating and treatment costs were made at 6 weeks, 3, 6 and 12 months from initial treatment.

**Fig. 3:**
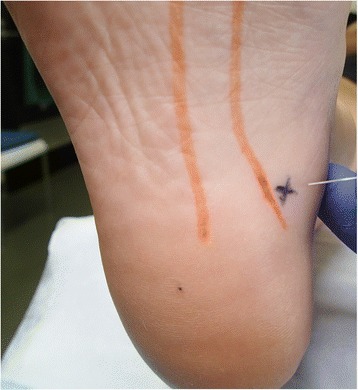
Plantar photograph of left foot illustrating the injection site used by Kim et al. [35] for management of plantar faciopathy. The ‘X’ marking represents the medial heel site used for the ultrasound-guided platelet-rich-plasma and prolotherapy injections, with the *orange lines* demarking the medial band of the plantar fascia

Kim et al. [35] compared the efficacy of autologous platelet-rich plasma with prolotherapy injections in 21 participants with chronic recalcitrant plantar fasciopathy aged from 19 to 57 years. Participants with odd participant sequence numbers were allocated to the prolotherapy group (11 participants), while participants with even sequence numbers were placed in the autologous platelet-rich plasmagroup (10 participants). The injection procedure was performed under aseptic conditions using a 22-gauge needle. Figure 3 illustrates the location of needle insertion through the medial heel, which was performed under ultrasound-guidance toward the target area. Approximately 2 mL of platelet-rich plasmaor prolotherapy solution was injected using a peppering technique, which involved a single skin portal followed by five penetrations of the fascia. Both interventions consisted of two injections at 2-weekly intervals. Final follow-up occurred between 10 and 28 weeks from initial treatment date, with all participants completing the follow-up with the exception of one in the platelet-rich plasmagroup. Ryan et al. [36] conducted a prospective case-series to evaluate the effectiveness of sonographically-guided prolotherapy injections in reducing pain in 20 participants (17 women) with chronic plantar fasciopathy. Outcome measures including the Foot Function Index (FFI) and VAS pain were recorded at baseline and the final treatment consultation, with a follow-up telephone interview conducted a mean of 11.8 months (range 6–20 months) following the participant’s final session.

**Fig. 4:**
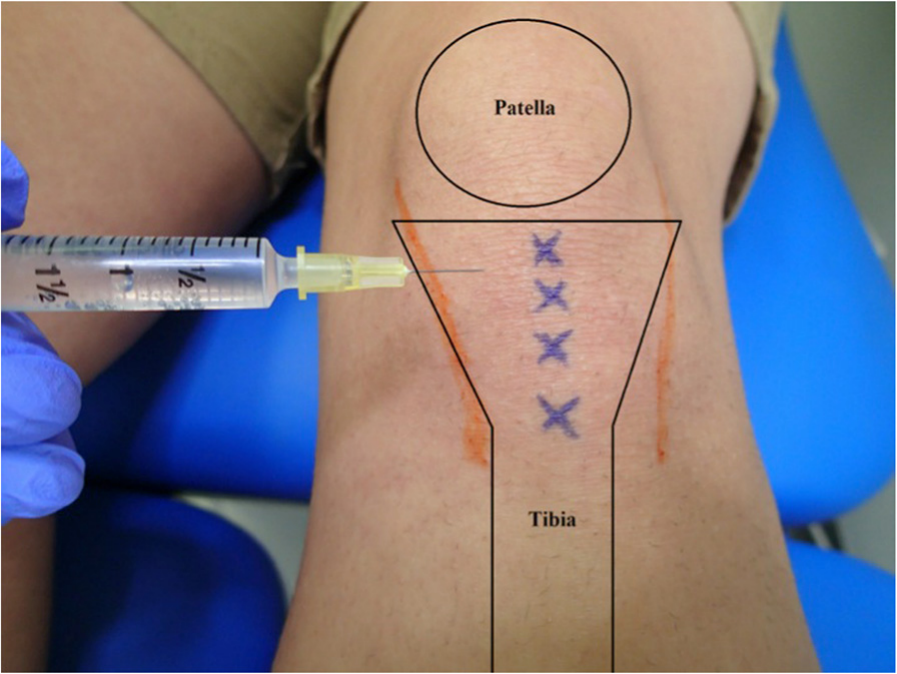
Anteroposterior photograph of knee illustrating injection points marked ‘X’ starting over the most distal area of pain on the tibial tuberosity and moving proximally in 1-cm increments to the most proximal painful point with pressure as described by Topol et al. [37]. The *orange lines* represent the attachment of the patellar tendon from the patella to the tuberosity or its fragments

In the only study included in this analysis that assessed prolotherapy injections for Osgood-Schlatter Disease, Topol et al. [37] performed a double-blinded randomised controlled trial involving 65 knees in 54 participants. Participants (mean age 13.3 years) were randomly allocated to receive prolotherapy injections (21 knees), lignocaine injections (22 knees) or supervised usual care (22 knees). Locations and technique for the lignocaine and prolotherapy injections can be seen in Fig. 4. Topol et al. [37] used the Nirschl Pain Phase Scale (NPPS), which is a 7-level measure of sports inhibition and sports-related symptoms. The threshold goals in the study were a NPPS score <4, where symptoms may be present however sport is uninhibited, or a NPPS score below 4 where asymptomatic sport occurs. This study assessed sport alteration and sport-related symptoms in adolescent athletes with Osgood-Schlatter Disease. All participants that still reported sports-related symptoms after 3 months, the point at which the actual injectant was revealed to the treating physician and participant, could then choose to receive prolotherapy injections. This was offered monthly until 12 months after either elimination of symptoms or plateau of improvement. Athletes were not required to receive dextrose injection if they were satisfied with their status at 3 months.

### Effects of interventions

#### Pooled standardised mean differences for short-, intermediate- and long-term data

**Fig. 5:**
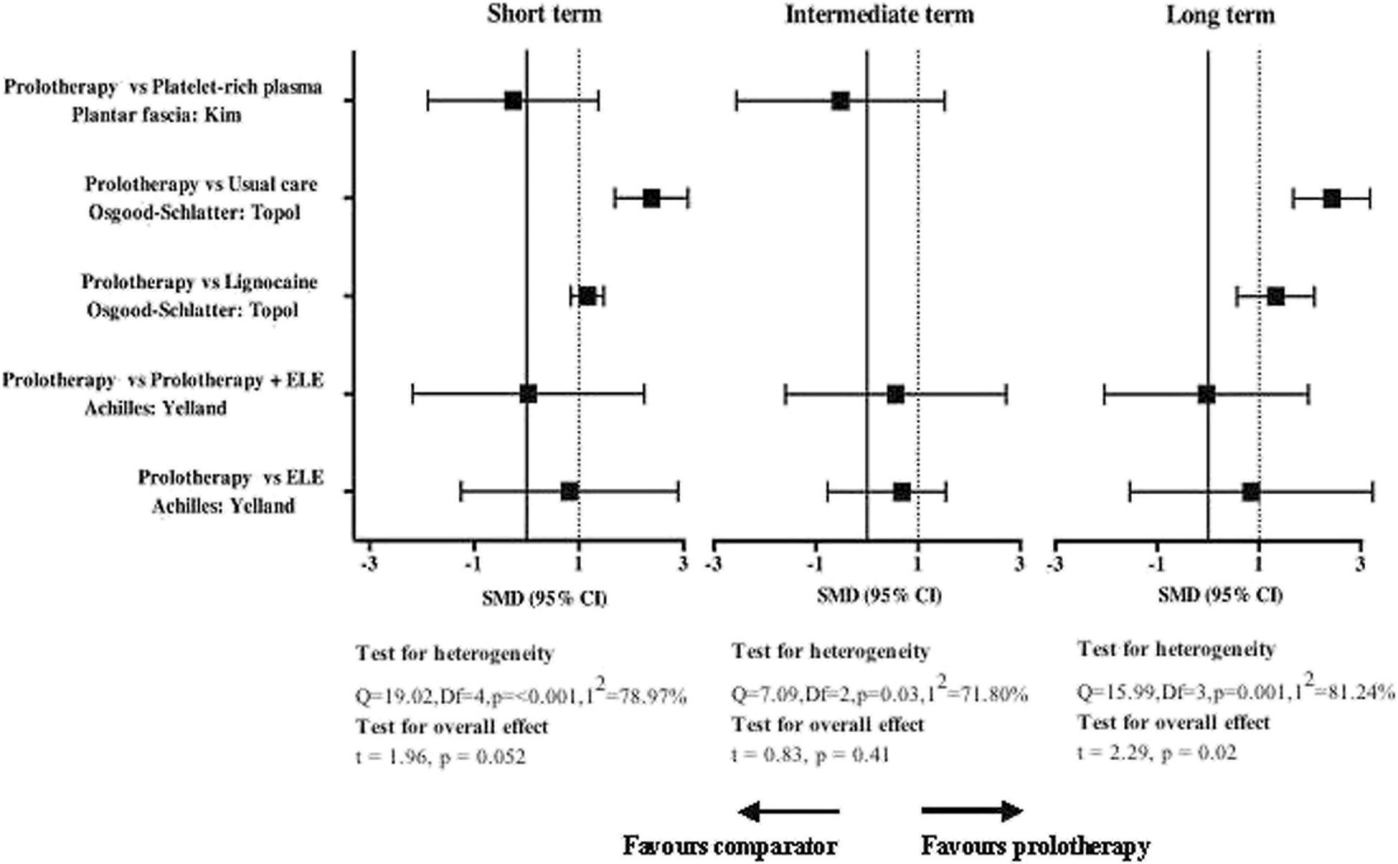
Standardised mean differences (SMD) for improved pain after prolotherapy vs comparator intervention for plantar fasciopathy, Osgood Schlatter disease and Achilles tendinopathy

Meta-analysis was only possible for three of the included studies. SMDs were calculated for Yelland et al. [34], Kim et al. [35] and Topol et al. [37] and are presented in the forest plots in Fig. 5. Substantial heterogeneity was present in the comparison between prolotherapy and other interventions for short- (*P* < 0.001, I^2^ = 79 %), intermediate- (*P* = 0.029, I^2^ = 72 %) and long-term data (*P* = 0.001, I^2^ = 81 %). A statistically significant total effect was found between prolotherapy and other intervention for long-term data (*P* = 0.024), with the total (random effects) SMD found to be 0.48 (0.15 to 2.03). Short-term data approached statistical significance (*P* = 0.052) with a total SMD of 0.38 (−0.01 to 1.48) while the intermediate-term total SMD of 0.47 (−0.55 to 1.33) was not found to be statistically significant (*P* = 0.410). Data from the remaining five studies could not be pooled due lack of a comparator group, and qualitative and quantitative analysis of all individual studies are discussed in the following pathology-specific sections.

#### Prolotherapy injections for treatment of Achilles tendinopathy

Five studies of moderate to good methodological quality involving 338 participants reported on management of painful Achilles tendinopathy with prolotherapy injections. The studies included: prolotherapy as the only intervention with no control group (Lyftogt [30], Lyftogt [31], Maxwell et al. [32], Ryan et al. [33]); and prolotherapy versus eccentric loading exercises versus combination of prolotherapy plus eccentric loading exercises (Yelland et al. [34]).

##### Pain

All five studies reported a significant reduction in pain following prolotherapy treatment. Lyftogt [30] reported a mean reduction in VAS pain score of 62.0 mm by final 16-week follow-up. In another study, Lyftogt [31] reported a mean reduction in VAS scores for three different prolotherapy regimens with results as follows: 60.2 mm for dextrose 20 %/lignocaine 0.1 %, 56.0 mm for dextrose 30 %/lignocaine 0.1 %/ropivacaine 0.1 % and 56.0 mm for dextrose 40 %/ropivacaine 0.1 % with a mean length of treatment 8.0, 7.6 and 8.7 weeks respectively. Not enough data was supplied to calculate the 95 % CIs for these studies. Maxwell et al. [32] reported a mean percentage reduction at final follow-up for VAS1 (pain during rest) of 88.2 % (*p* < 0.001), for VAS2 (pain during normal daily activity) of 84.0 % (*p* < 0.001), and for VAS3 (pain during or after sporting activity) of 78.1 % (*p* < 0.001) following sonographically-guided intratendinous prolotherapy injections. The same injection regimen was repeated 3 years later in a study by Ryan et al. [33] who reported significant improvement in pain scores for both midportion Achilles tendinopathy [VAS1 90.3 % (*p* < 0.001), VAS2 81.07 (*p* < 0.001), VAS3 76.3 % (*p* < 0.001)] and insertional Achilles tendinopathy [VAS1 91.5 % (*p* < 0.001), VAS2 80.5 % (*p* < 0.001), VAS3 74.6 % (*p* < 0.001)] participants from baseline to final follow-up.

Yelland et al. [34] were contacted for unpublished VAS pain data used in the study from which the SMD between intervention groups was calculated. As can be seen in the forest plot in Fig. 5, a large effect was seen in favour of prolotherapy vs eccentric loading exercises for short- (SMD 0.82, 95 % CI −2.18 to 2.25), intermediate- (1.26, 95 % CI −0.77 to 1.54) and long-term pain (0.84, 95 % CI −1.54 to 3.23). For prolotherapy compared to combination (prolotherapy plus eccentric loading exercises) therapy, a small and moderate effect was found in favour of prolotherapy for short- (0.04, −2.18 to 2.25) and intermediate-term (0.56, 95 % CI −1.60 to 2.72) pain, however a small effect in favour of combination therapy was seen long-term (−0.04, 95 % CI −2.04 to 1.97). It should be noted that although the SMDs for VAS pain favoured prolotherapy and combination therapy over eccentric loading exercises, none of these differences were found to be statistically significant. Similarly, Yelland et al. [34] reported decreases in pain scores from baseline for eccentric loading exercises, which was significantly less than for prolotherapy at 6 months and for combined treatment at 12 months. However, more importantly, the difference between treatment groups over time was not significant overall.

##### Pathology or location-specific outcome measures

At 12 months, Yelland et al. [34] reported that the proportions achieving the minimum clinically important change for VISA-A were 73 % for eccentric loading exercises, 79 % for prolotherapy and 86 % for combined treatment. Mean improvements in VISA-A scores at 12 months were 23.7 points for eccentric loading exercises, 27.5 points for prolotherapy and 41.1 points for combined treatment. At 6 weeks and 12 months, these increases were significantly less for eccentric loading exercises than for combined treatment. Compared with eccentric loading exercises, reductions in stiffness and limitation of activity occurred earlier with combined treatment.

##### Satisfaction

Lyftogt [30] reported 88 % satisfaction for the single cohort at 16-week follow-up, while the satisfaction for each treatment group in Lyftogt [31] was: A) 96 % at mean 21-month follow-up, B) 88 % at mean 12-month follow-up, and C) no data supplied. Twenty of 32 participants were asymptomatic at 12 months after treatment in the study by Maxwell et al. [32], with 19 participants giving a satisfaction level of 95–100 %. Only one participant had moderate symptoms and described only 50 % satisfaction level. Yelland et al. [34] found the percentage of participants reporting “satisfaction” or “extreme satisfaction” with treatment at 12 months was 50 % for eccentric loading exercises, 69 % for prolotherapy and 71 % for combined treatment, however the differences between groups was not statistically significant.

##### Sonographic measurements of tendon thickness, size of hypoechoic region and intratendinous tear size

Maxwell et al. [32] reported the mean Achilles tendon thickness decreased from 11.7 to 11.1 mm (*p* < 0.007) following prolotherapy treatment. The number of tendons with anechoic clefts or foci was reduced by 78 %. Echogenicity improved in 6 tendons (18 %), but was unchanged in 27 tendons (82 %). Neovascularity was unchanged in 11 tendons (33 %) but decreased in 18 tendons (55 %); no neovascularity was present before or after treatment in the remaining tendons. Similarly, Ryan et al. [33] also noted a reduction in painful symptoms corresponded with improvements in some aspects of the sonographic appearance of the Achilles tendon. There were no differences in the recorded thicknesses of the Achilles tendon at either injury site. There was a greater reduction in the number of participants with both grade 3 or 2 hypoechogenicity and neovascularization in the midportion group at post-test compared with the insertional group.

##### Adverse events

Lyftogt [30], Lyftogt [31] and Ryan et al. [33] did not provide information on post-intervention complications. Maxwell et al. [32] reported no adverse effects or complications following prolotherapy injections, and concluded hyperosmolar dextrose has “an excellent safety profile”. Yelland et al. [34] was the only study to report an adverse effect; in which one participant in the eccentric loading exercises group had a partial calf tear while playing tennis. An independent sports physician did not attribute this to the eccentric loading exercises program.

#### Prolotherapy injections for treatment of plantar fasciopathy

Two studies of moderate to good methodological quality involving 41 participants reported on management of painful plantar fasciopathy with prolotherapy injections. The studies included: prolotherapy versus platelet-rich plasma (Kim et al. [35]); and prolotherapy as the only intervention with no control group (Ryan et al. [36]).

##### Pain

Both Kim et al. [35] and Ryan et al. [36] reported a significant reduction in painful symptoms following prolotherapy injections in participants with painful plantar fasciopathy. Figure 5 shows the SMD for improvement in pain after prolotherapy vs platelet-rich plasma injections, with Kim et al. [35] reporting a small short-term (SMD −0.26, 95 % CI −1.88 to 1.37) and moderate intermediate-term (−0.52, 95 % CI −2.56 to 1.52) effect in favour of platelet-rich plasma. However, both prolotherapy and platelet-rich plasma interventions had significant improvements on pain levels, with a 17.1 % and 29.7 % improvement at 28 weeks respectively. Ryan et al. [36] found a significant decrease (*p* < 0.001) in all mean VAS items from pre-test to post-test: VAS1 (36.8 to 10.3), VAS2 (74.7 to 25.0) and VAS3 (91.6 to 38.7) with no significant changes at the final follow-up interview.

##### Pathology or location-specific outcome measures

Kim et al. [35] found the mean Foot Function Index total and subcategory score improvements were greater in the platelet-rich plasma group (30.4 %) compared with the prolotherapy group (15.1 %), however no statistically significant differences were noted at any follow up. In the pain and disability subcategories, both groups showed significant improvements at the last re-evaluation.

##### Adverse events

Ryan et al. [36] reported no complications from prolotherapy injections into the plantar fascia. Kim et al. [35] found most participants in both intervention groups reported local pain or discomfort that started on the day of injection and subsided gradually, however no other complications of either injection therapy were reported.

#### Prolotherapy injections for treatment of Osgood-Schlatter disease

One trial of moderate to good methodological quality involving 54 participants reported on management of painful Osgood-Schlatter disease with prolotherapy injections. The study included prolotherapy versus usual care versus lignocaine injections (Topol et al. [37]).

##### Pain

SMD calculations of the results reported by Topol et al. [37] found a large effect size in favour of prolotherapy versus usual care or lignocaine injections for short-term (prolotherapy versus usual care SMD 2.39, 95 % CI 1.69 to 3.08; prolotherapy versus lignocaine 1.16, 95 % CI 0.85 to 1.48) and long-term pain levels (prolotherapy versus usual care 2.43, 95 % CI 1.67 to 3.18; prolotherapy versus lignocaine 1.33, 95 % CI 0.57 to 2.09) – illustrated in Fig. 5. Figure 5 also shows that the prolotherapy intervention had a large positive effect when compared to usual care and lignocaine, with the lower confidence interval not crossing zero (the line of no effect) for both short- and long-term outcomes.

##### Return to activity

Topol et al. [37] reported unaltered sport was more common in both prolotherapy-treated (21 of 21 versus 13 of 22; *p* = 0.001) and lignocaine-treated (20 of 22 versus 13 of 22; *p* = 0.034) knees when compared with usual care at 3 months In addition, asymptomatic sport was more frequent in the prolotherapy-treated knee group than either lignocaine-treated (14 of 21 versus 5 of 22; *p* = 0.006) or usual care-treated (14 of 21 versus 3 of 22; *p* < 0.001) knee groups. At 1 year, asymptomatic sport was more common in the prolotherapy-treated knee group than in groups that had knees treated with only lignocaine (32 of 38 versus 6 of 13; *p* = 0.024) or only usual care (32 of 38 versus 2 of 14; *p* < 0.001).

##### Adverse events

Topol et al. [37] did not provide information on post-intervention complications for any of the participant groups, however the study reported 100 % of the prolotherapy intervention group participants achieved unaltered sport by the final blinded period follow-up without missing a monthly injection, indicating no complications occurred following prolotherapy injections in the trial.

## Discussion

Data was only able to be pooled and analysed for the three studies with multiple interventions, and while a statistically significant overall difference between prolotherapy and other interventions was found for short- and long-term SMD data, the small sample size and testing of various pathologies reduces the weight of these findings. Considerable variance between the studies was determined for short-, medium- and long-term data (I^2^ = 72–81 %), a consequence of both clinical and methodological diversity among the studies. The assessment of outcome was hampered by the variety and quality of the outcomes recorded in the eight studies that were included. The VAS for assessment of pain was the most frequently used outcome, either generally or specific to rest and activity (VAS1; VAS2; VAS3). Pain was also measured through the VISA-A and NPPS measures, further complicating comparisons between studies. Ryan et al. [33] successfully built on the previous research of Maxwell et al. [32] by using the same pain VAS scores and sonographic Achilles tendon measurements, however these were the only repeated outcome measurements analysed in this review.

All five studies included in this review (Lyftogt [30]; Lyftogt [31]; Maxwell et al. [32]; Ryan et al. [33]; Yelland et al. [34]) showed moderate evidence of improved Achilles tendinopathy pain, function and participant satisfaction with corresponding sonographic measurements following prolotherapy intervention. Yelland et al. [34] was the only study to compare prolotherapy injections with the Achilles tendinopathy reference standard treatment of eccentric loading exercises and reported superior pain and stiffness reduction at 6-months, with the benefit of minimal effort required of the participant, and the technique being a time-efficient approach. Yelland et al. [34] also found combination therapy of eccentric loading exercises plus prolotherapy was the most beneficial intervention for improvement in VISA-A and VAS pain scores at 12-months. No adverse events following prolotherapy for management of Achilles tendinopathy were reported in any of the studies included in our analysis. Despite the positive overall results presented in these studies the heterogeneity in treatment approaches and control groups as well as poor overall methodology prohibited an extensive meta-analysis and significantly limits the strength of conclusions for this review.

The two studies relating to chronic plantar fasciopathy included in this review indicate that injections of dextrose and lignocaine reduces pain in the majority of participants. Eighty percent of participants undergoing ultrasound-guided treatment in the study by Ryan et al. [36] reported good to excellent outcomes in pain reduction at the cessation of their treatment. The case-series design and the lack of control group in this study limit the evidence of the treatment effect, however the results are promising for patients experiencing chronic plantar fasciopathy unresponsive to conservative treatment. Kim et al. [35] compared prolotherapy with platelet-rich plasma; an increasingly popular treatment that, like prolotherapy, is hypothesised to stimulate processes associated with healing. The results of Kim et al. [35] indicate that there is no clear advantage or disadvantage in using either prolotherapy or platelet-rich plasma for management of chronic plantar fasciopathy, with improvements in both pain and function seen in both intervention techniques. Platelet-rich plasma injections did result in better outcomes in FFI total scores and the pain subcategory, although no significant difference was noted between groups and both treatments markedly reduced pain within a few months after injection indicating that both may be an effective treatment option for chronic plantar fasciopathy. However, these findings need to be considered in the context that a true control group (i.e. placebo) was not included, so it is not known how effective they are relative to no treatment. Ryan et al. [36] and Kim et al. [35] both reported no adverse events following prolotherapy or platelet-rich plasma injections.

The single trial that assessed prolotherapy for treatment of Osgood-Schlatter disease included in this review found that prolotherapy was superior to usual care for improving unaltered sport and asymptomatic sport outcomes. Topol et al. [37] reported lignocaine injections resulted in similar improvement to prolotherapy in unaltered sport; with 90 and 100 % of participants reporting unaltered sport at 3-months for lignocaine and prolotherapy-treated groups, respectively. However Topol et al. [37] found evidence that prolotherapy was significantly superior to lignocaine injection for improvement in asymptomatic sport, with 67 % of the prolotherapy group and 23 % of lignocaine group reporting complete asymptomatic sport at 3-months. The duration of surveillance was a 3-month blinded period, which was sufficient to see immediate improvements in outcome, but further long-term follow up would have been more beneficial in evaluating the effectiveness between groups. Adverse events were not reported in this study.

This systematic review has several limitations. Firstly, SMDs were only calculated for three studies; it was not possible to pool SMDs in the five remaining studies as no comparator group was present. Secondly, the review was restricted to prospective studies, so only three pathologies were included; Achilles tendinopathy, plantar fasciopathy and Osgood-Schlatter disease. This may have excluded useful clinical information and by excluding studies assessing prolotherapy for ligament disorders a large body of data was not considered. However, the pathomechanics and prolotherapy mechanism of action differ greatly for ligament pathologies and their inclusion may have reduced the specificity and generalisability of our systematic review. Thirdly, the prolotherapy regimen, including concentration of dextrose, agent and concentration of additional local anaesthetic/s, and the frequency, volume and total number of injections varied significantly between studies. Only a few of the variety of prolotherapy interventions have been evaluated and the interventions that have been evaluated assessed different anatomical and physiological pathologies, thus no conclusive comparisons can be made between them. Fourthly, the heterogeneity of study populations was also generally poor, particularly regarding sex of participants, with the majority of studies being heavily skewed towards one sex type that created large potential for confounding. Finally, the greatest limitation of the study was the lack of high quality randomised controlled trials available in the literature; in our review, the conclusions about prolotherapy injections in the lower limb were made on the basis of only three randomised controlled trials. To address these limitations, it is essential that future studies should analyse prolotherapy for tendinopathy and fasciopathy using high-quality randomised controlled trial methodology. Future research should also consider inclusion of other musculoskeletal disorders, recruitment of large sample sizes, standardisation of co-interventions, long-term follow-up, and systematic reporting of adverse events are needed.

## Conclusions

This systematic review identified eight studies that reported on the effectiveness of prolotherapy for treatment of lower-limb tendinopathies and fasciopathies and found that there is limited evidence to support prolotherapy for the treatment of these pathologies. Of the studies that were reviewed, only a small proportion were randomised controlled trials, with the majority of studies employing a prospective case-series design using a relatively small sample size and no control or usual care group. With these limitations in mind, analysis of the three randomised controlled trials that were included in this review suggests that prolotherapy injections may be superior or at least comparable to eccentric loading exercises for Achilles tendinopathy, platelet-rich plasma for plantar fasciopathy, and usual care or lignocaine injections for Osgood-Schlatter disease. No study in this review reported an adverse event following prolotherapy injection. Further research using large, methodologically-sound randomised controlled trials is needed to support the use of prolotherapy for lower-limb tendinopathies and fasciopathies.

## Additional files

Additional file 1:
**Included studies.** (DOCX 14 kb) Additional file 2:
**Reasons for the exclusion of studies after full text assessment.** (DOCX 12 kb)
