# Erratum to: Effectiveness and safety of prolotherapy injections for management of lower limb tendinopathy and fasciopathy: a systematic review

**DOI:** 10.1186/s13047-015-0117-2

**Published:** 2015-11-04

**Authors:** Lane M. Sanderson, Alan Bryant

**Affiliations:** School of Surgery, University of Western Australia, Perth, Australia; M422 UWA Podiatric Medicine, 35 Stirling Highway, Crawley, 6009 WA Australia

## Erratum

Unfortunately the original version of this article [1] contained an error. The subheadings within the abstract were included incorrectly. The correct subheadings and complete abstract can be found corrected below:

## Abstract

### Background

The aim of this review was to identify and evaluate existing research to determine the clinical effectiveness and safety of prolotherapy injections for treatment of lower limb tendinopathy and fasciopathy.

### Methods

Nine databases were searched (Medline, Science Direct, AMED, Australian Medical Index, APAIS-Health, ATSIhealth, EMBASE, Web of Science, OneSearch) without language, publication or data restrictions for all relevant articles between January 1960 and September 2014. All prospective randomised and non-randomised trials, cohort studies, case-series, cross-sectional studies and controlled trials assessing the effectiveness of one or more prolotherapy injections for tendinopathy or fasciopathy at or below the superior aspect of the tibia/fibula were included. Methodological quality of studies was determined using a modified evaluation tool developed by the Cochrane Musculoskeletal Injuries Group. Data analysis was carried out to determine the mean change of outcome measure scores from baseline to final follow-up for trials with no comparative group, and for randomised controlled trials, standardised mean differences between intervention groups were calculated. Pooled SMD data were calculated where possible to determine the statistical heterogeneity and overall effect for short-, intermediate- and long-term data. Adverse events were also reported.

### Results

Two hundred and three studies were identified, eight of which met the inclusion criteria. These were then grouped according to tendinopathy or fasciopathy being treated with prolotherapy injections: Achilles tendinopathy, plantar fasciopathy and Osgood-Schlatter disease. The methodological quality of the eight included studies was generally poor, particularly in regards to allocation concealment, intention to treat analysis and blinding procedures. Results of the analysis provide limited support for the hypothesis that prolotherapy is effective in both reducing pain and improving function for lower limb tendinopathy and fasciopathy, with no study reporting a mean negative or non-significant outcome following prolotherapy injection. The analysis also suggests prolotherapy injections provide equal or superior short-, intermediate- and long-term results to alternative treatment modalities, including eccentric loading exercises for Achilles tendinopathy, platelet-rich plasma for plantar fasciopathy and usual care or lignocaine injections for Osgood-Schlatter disease. No adverse events following prolotherapy injections were reported in any study in this review.

### Conclusions

The conclusions of this review were derived from the best available scientific evidence. It is intended that the results of this study will assist clinical decision-making by practitioners. The results of this review found limited evidence that prolotherapy injections are a safe and effective treatment for Achilles tendinopathy, plantar fasciopathy and Osgood-Schlatter disease, however more robust research using large, methodologically-sound randomised controlled trials is required to substantiate these findings.
