# Supplementary material for: Effectiveness and safety of prolotherapy injections for management of lower limb tendinopathy and fasciopathy: a systematic review
Source: J Foot Ankle Res. 2015 Oct 20;8:57. doi: 10.1186/s13047-015-0114-5 (PMC4617485; doi:10.1186/s13047-015-0114-5)
Supplement: Additional file 1: — Included studies. (DOCX 14 kb) [file 13047_2015_114_MOESM1_ESM.docx]

**Additional data file 1: included studies**

| Authors | Title | Year | Volume(issue): page numbers | *Journal* |
| --- | --- | --- | --- | --- |
|  |  |  |  |  |
| Kim et al | Autologous platelet-rich plasma versus dextrose prolotherapy for the treatment of chronic recalcitrant plantar fasciitis. | 2010 | **16**(12): 1285–1290 | *Journal of Alternative and Complementary Medicine* |
| Lyftogt | Prolotherapy and Achilles tendinopathy: A prospective pilot study of an old treatment. | 2005 | Pg 16-19 | *Australasion Musculoskeletal Medicine* |
| Lyftogt | Subcutaneous prolotherapy for Achilles tendinopathy: The best solution? | 2007 | Pg 107-109 | *Australasion Musculoskeletal Medicine* |
| Maxwell et al | Sonographically guided intratendinous injection of hyperosmolar dextrose to treat chronic tendinosis of the Achilles tendon: a pilot study. | 2007 | **189**(4): W215-220 | *American Journal of Roentgenology* |
| Ryan et al | Favourable outcomes after sonographically guided intratendinous injection of hyperosmolar dextrose for chronic insertional and midportion Achilles tendinosis. | 2010 | **194**:1047-1053 | *American Journal of Roentgenology* |
| Ryan et al | Sonographically guided intratendinous injections of hyperosmplar dextrose/ lignocaine: a pilot study for the treatment of chronic plantar fasciitis. | 2009 | **43**: 303-306 | *British Journal of Sports Medicine* |
| Topol et al | Hyperosmolar dextrose injection for recalcitrant Osgood-Schlatter disease. | 2008 | **87**(11): 890-902 | *American Journal of Physical Medicine and Rehabilitation* |
| Yelland et al | Prolotherapy injections and eccentric loading exercises for painful Achilles tendinosis: a randomised trial. | 2011 | **45**: 421-428 | *British Journal of Sports Medicine* |
|  |  |  |  |  |
