# Supplementary material for: Effectiveness and safety of prolotherapy injections for management of lower limb tendinopathy and fasciopathy: a systematic review
Source: J Foot Ankle Res. 2015 Oct 20;8:57. doi: 10.1186/s13047-015-0114-5 (PMC4617485; doi:10.1186/s13047-015-0114-5)
Supplement: Additional file 2: — Reasons for the exclusion of studies after full text assessment. (DOCX 12 kb) [file 13047_2015_114_MOESM2_ESM.docx]

**Additional data file 2: reasons for the exclusion of studies after full text assessment**

| **Principal author** | **Year** | **Reason for exclusion** |
| --- | --- | --- |
|  |  |  |
| Coombes | 2010 | Review only |
| DeChellis | 2011 | Review only |
| Dumais | 2012 | Did not assess fasciopathy or tendinopathy |
| Goswami | 2012 | Patient education article |
| Gracer | 2005 | Theoretical study |
| Gross | 2013 | Review only |
| Harvey | 2007 | Review only |
| Hauser | 2011 | Retrospective study |
| Hauser | 2012 | Did not assess fasciopathy or tendinopathy |
| Hauser | 2012 | Combined prolotherapy with human growth hormone |
| Karam | 2012 | Did not assess fasciopathy or tendinopathy |
| Lakshmi | 2011 | Did not assess fasciopathy or tendinopathy |
| Lyftogt | 2006 | Did not assess fasciopathy or tendinopathy |
| Martins | 2012 | Non-human (rat) participants |
| Molloy | 2012 | Review only |
| Padhier | 2011 | Did not assess fasciopathy or tendinopathy |
| Sweeting | 2011 | Review only |
| Tsatsos | 2002 | Review only |
|  |  |  |
|  |  |  |
